# Harmonizing the CBCL and SDQ ADHD scores by using linear equating, kernel equating, item response theory and machine learning methods

**DOI:** 10.3389/fpsyg.2024.1345406

**Published:** 2024-07-10

**Authors:** Miljan Jović, Maryam Amir Haeri, Andrew Whitehouse, Stéphanie M. van den Berg

**Affiliations:** ^1^Department of Learning, Data Analytics and Technology, University of Twente, Enschede, Netherlands; ^2^Telethon Kids Institute, University of Western Australia, Perth, WA, Australia

**Keywords:** data harmonization, test equating, machine learning, IRT, linear equating, kernel equating, ADHD

## Abstract

**Introduction:**

A problem that applied researchers and practitioners often face is the fact that different institutions within research consortia use different scales to evaluate the same construct which makes comparison of the results and pooling challenging. In order to meaningfully pool and compare the scores, the scales should be harmonized. The aim of this paper is to use different test equating methods to harmonize the ADHD scores from Child Behavior Checklist (CBCL) and Strengths and Difficulties Questionnaire (SDQ) and to see which method leads to the result.

**Methods:**

Sample consists of 1551 parent reports of children aged 10-11.5 years from Raine study on both CBCL and SDQ (common persons design). We used linear equating, kernel equating, Item Response Theory (IRT), and the following machine learning methods: regression (linear and ordinal), random forest (regression and classification) and Support Vector Machine (regression and classification). Efficacy of the methods is operationalized in terms of the root-mean-square error (RMSE) of differences between predicted and observed scores in cross-validation.

**Results and discussion:**

Results showed that with single group design, it is the best to use the methods that use item level information and that treat the outcome as interval measurement level (regression approach).

## 1 Introduction

When researchers work with data from different institutions, they often encounter situations where different scales are used for the evaluation of the same construct. This makes pooling of data and comparison of the results challenging. Nevertheless, combining data from different groups of participants who filled in different questionnaires is often necessary to obtain (a) sufficiently large sample sizes, (b) to be able to make comparisons across subpopulations, or (c) to increase generalizability and validity of research results (Smith-Warner et al., 2006; Thompson, 2009; Fortier et al., 2010, 2011; Hamilton et al., 2011; van den Berg et al., 2014).

Two mental health instruments that are widely used by different institutions for assessing the same constructs are the Child Behavior Checklist (CBCL) and the Strengths and Difficulties Questionnaire (SDQ). Both assess mental health problems among children and adolescents, but they differ both quantitatively (different number of items) and content wise (e.g., different phrasing of items). The CBCL consists of 113 items and operationalizes childhood problem behavior on eight subscales/dimensions (social withdrawal, somatic complaints, anxiety/depression, social problems, thought problems, attention problems, delinquent behavior, and aggressive behavior; Achenbach et al., 1991; Achenbach and Ruffle, 2000). The SDQ consists of 25 items equally divided across five scales, also called dimensions (Emotional, Conduct, Hyperactivity, Peer, and Prosocial problems; Goodman, 1997, 2001).

Both instruments are already well-established and widely used for assessing psychopathology in general, but also for assessing specific mental health problems (Achenbach, 1991; Allen and Prior, 1995; Caspi et al., 1995; Muris et al., 2003; Ortuno-Sierra et al., 2015).

One relatively common mental health problem in children is Attention-Deficit Hyperactivity Disorder (ADHD). Both CBCL and SDQ contain items that address ADHD-related symptoms and various research studies proved that both of those instruments perform well in the context of screening for ADHD problems (Chen et al., 1994; Algorta et al., 2016; Hall et al., 2019).

Even though they are both valid, there are differences in the content and number of items. SDQ has a hyperactivity scale that also includes items that measure concentration problems (SDQ; 5 ADHD items in total), while CBCL is more focused on attention problems but also contains hyperactivity items (CBCL; 11 ADHD items in total). Both measure ADHD in broader sense, but they do not completely overlap. Those differences make it difficult to compare scores on SDQ and CBCL scales directly, as they have different distributions (e.g., different means and different variance). In order to make the scores obtained by those instruments comparable, it is necessary to harmonize them, that is, to put them on the same scale. Such a scale could be the SDQ scale, where CBCL scores are transformed in some way into an SDQ scale, or vice versa. Alternatively, both SDQ and CBCL scale scores could be translated into a third, normalised scale score, with for instance mean 0 and variance 1.

There are different methodologies that can be used for data harmonization, and the most common one is test equating, also known as test linking or scaling (Mislevy, 1992; Holland et al., 2006; Kolen and Brennan, 2014). It is applied mostly in the context of educational measurement, where the test scores from one exam need to be harmonized somehow with test scores from a similar but different exam.

The type of method used for test equating depends on what information is available or used. For example, if we only have test scores on exam A in pupils from school I and test scores on exam B in pupils from school II, we can either use only the mean test scores, we use both means and standard deviations, or we use the entire distributions in terms of quantiles. The respective methods that are based on these statistics are mean equating, linear equating and equipercentile equating. The strong assumption in these methods is that the scores provide all the necessary information (sufficiency) and that the two tests measure exactly the same trait, conceptually. Although reasonable for exam versions in education, in the context of psychopathology this may be too strong an assumption.

In the case if scales do not measure exactly the same thing, we need data to link those two scales. In that case, we need either at least some common items in both scales (Common Items study design with so-called anchor items), or the same group of persons that filled in both scale versions (Common Persons study design, a.k.a. single group design).

In Common Items design, there are two different samples of participants. One sample filled in scale A while another filled in scale B. Majority of items in those scales are different, but there is a certain number of items that is common for both scales. Accordingly, those overlapping (common) items that are present in both scales can be used to obtain harmonized scores.

Another design is Common Persons (single group) design in which we have only one sample of participants, but they filled in both scales at the same time. Since we have responses of all participants on both scales, we can use them to harmonize the scores.

This type of information (raw data at the item level), when it is available, could be used in a powerful way by implementing Item Response Theory (IRT) models. These models take into account not only differences between test takers, but also between the items, for instance their relative difficulty (van den Berg et al., 2014; Jabrayilov et al., 2016; Sansivieri et al., 2017; Jović et al., 2022; Mansolf et al., 2022).

Still, the basic unidimensional IRT approach includes the assumption that exactly the same trait is measured. If we allow for the possibility that the scales only partly overlap, in that the constructs that are measured are only correlated, we could use either a more complex IRT model (more complex than Rasch models), or try a whole bunch of other methods. Rasch model is a simple IRT model in which participants` response to an item is determined by the latent trait level of the participant and difficulty/threshold parameter of the item. Threshold parameter is defined as the point on the latent trait continuum where the response probability for two adjacent response categories is equal (Wetzel and Carstensen, 2014). In more complex IRT models (e.g., Generalized Partial Credit Model), participants` responses are determined not only by their latent trait value and difficulty of the item, but also by discrimination parameter of the item (refers to the strength of the relationship between trait level and participants` responses on the item; see Embretson and Reise (2000) for more details). For example, van den Berg et al. (2014) used Generalized Partial Credit Model to harmonize neuroticism and extraversion scores, while Jović et al. (2022) used it to harmonize anxiety/depression and ADHD scores of CBCL and SDQ scales. Mansolf et al. (2022) used IRT to harmonize Internalizing, Externalizing, and Total Problems domains from CBCL and SDQ. Recently there has been attention for methods based on the machine learning (ML) literature. For example, Jiang et al. (2023) used ensemble learning and their results showed that ML based equating outperformed Mean, Linear and Equipercentile equating methods both in simulation and empirical studies (educational assessment). Tsutsumi et al. (2021) successfully combined deep learning and IRT for data harmonization of both simulated and actual datasets.

### 1.1 Existing research on CBCL and SDQ data harmonization

In the past few years, there were a few interesting research studies that aimed to harmonize CBCL and SDQ data and they used different methodologies for data harmonization. Stevens et al. (2021) harmonized CBCL and SDQ total scores on a sample of 284 high-risk youth in a residential care facility. They used equipercentile equating. Mansolf et al. (2022) harmonized Internalizing, Externalizing, and Total Problems domains separately on a sample of 1,500 participants from general population between 2 and 17 years old. They used both equipercentile equating and IRT. They evaluated the quality of harmonization using the correlation between harmonized and observed scores: these were all higher than 0.82, except for Externalizing in the school-aged samples, which reached a minimum of about 0.75 for females ages 12–17 (Mansolf et al., 2022).

Jović et al. (2022) focused on harmonizing ADHD and anxiety/depression scores obtained by using CBCL and SDQ on a sample of 1,330 participants between 10 and 11.5 years old from Australia. Authors used IRT to harmonize CBCL and SDQ.

In all three research studies, the participants filled in both CBCL and SDQ scales, which is referred to as a common persons or single-group design, which is, according to Dorans (2007), ideal design for test linking.

They all had different samples. Stevens et al. (2021) used a high-risk population, while Mansolf et al. (2022) and Jović et al. (2022) harmonized data on a general population. They used different harmonization methods, equipercentile equating (Stevens et al., 2021) and IRT (Jović et al., 2022), while Mansolf et al. (2022) compared the results of both of those approaches. Also, they all harmonized CBCL and SDQ on a different level of granularity. Stevens et al. (2021) and Mansolf et al. (2022) harmonized externalising, internaling and total scores, while Jović et al. (2022) focused on more specific subscales (Anxiety/Depression and Hyperactivity/attention problems). In sum, it is unknown which harmonization method works the best in the case of CBCL and SDQ while harmonizing hyperactivity/attention problems.

### 1.2 The aim of the research

This study focuses on finding the most accurate approach for harmonizing hyperactivity/attention problem scores obtained by CBCL and SDQ scales. Our aim is to try out different data harmonization methods (that have different levels of complexity, different underlying assumptions and limitations) to see if there is one particular method that works best. We define best in the sense that a method helps us to put SDQ and CBCL on the same scale. For instance, the method should be able to translate a child’s score based on SDQ items into a CBCL-like score, so that the child’s level of ADHD related problems can be compared to those of its peers that only have CBCL item scores.

As mentioned above, the performance of the harmonization methods largely depends on the conceptual overlap between scales. If they completely overlap, the methods that use only mean and standard deviation or percentiles should be adequate enough. If, at the other hand, scales that we want to harmonize measure completely different constructs, we will need as much information as we can get. In the field of psychopathology it is hard to expect complete overlap between scales. Particularly a construct like ADHD where both attention problems and hyperactivity play a role, and different questionnaires put different emphases on these subdimensions. To see what works best, we will try out different methods of test equating, and compare their performance.

## 2 Methodology

### 2.1 Scales

The CBCL consists of 113 items and operationalizes childhood behavior on eight subscales/dimensions (social withdrawal, somatic complaints, anxiety/depression, social problems, thought problems, attention problems, delinquent behavior, and aggressive behavior; Achenbach et al., 1991; Achenbach and Ruffle, 2000). We used the attention problems subscale that includes both hyperactivity and attention problems.

The SDQ consists of 25 items equally divided across five scales, also called *dimensions* (Emotional, Conduct, Hyperactivity, Peer, and Prosocial problems; Goodman, 1997, 2001) and it is used for children aged 3–16 years. We used the hyperactivity-inattention scale that also includes items related to concentration problems.

### 2.2 Data collection design

Both CBCL and SDQ were administered to the same group of participants in the Raine study (McKnight et al., 2012). Accordingly, the Single-Group Design (Common Persons) was used to harmonize data in this study because we had responses of the same group of participants on both of scales. In the Single-Group Design, different scales that measure the same construct are administered to the same sample of participants. Different scales are filled in by the participants at the same time, so we assume that there were no changes in the measured construct that can affect the scores on different scales.

### 2.3 Sample

The Raine study is a prospective cohort of children that begun in 1989 and included 2,900 randomly assigned pregnant women who attended the public antenatal clinic at King Edward Memorial Hospital (KEMH; Perth, Western Australia) and nearby private clinics between May 1989 and November 1991 (Newnham et al., 1993; Chivers et al., 2010; Howard et al., 2011; McKnight et al., 2012). Those women completed questionnaires at 18 and 34 weeks of gestation, and follow-up investigations took place at birth, and at 1, 2, 3, 5, 8, 10, 14, 17, 18, and 20 years (Howard et al., 2011; McKnight et al., 2012). The study had two main aims: to investigate the hypothesis that complications of pregnancy might be prevented by frequent ultrasound scans and to develop a long-term cohort to study the role that early life events have on later health (McKnight et al., 2012). The subset of the dataset that we used for this study consists of both the CBCL and SDQ parent-filled questionnaires of 2,861 children (‘Generation 2’) aged between 10 and 11.5 years (1,417 girls, 1,444 boys). The 1991 Aseba version for the CBCL (age 4–18) by Achenbach (1991) and the 1997 SDQ version by Goodman (1997) were used. In the CBCL, the item scores consisted of either 0 – not true, 1 - omewhat/sometimes true, or 2 - very true/often true. In the SDQ, the responses are 0 – not true. 1 – somewhat true and 2 – certainly true. CBCL and SDQ data were collected at the same time. In this research, we used a subsample of 1,551 children whose mother provided responses on all attention problems/hyperactivity items (complete cases only). Which means only participants with complete answers on all 11 CBCL and 5 SDQ attention problems/hyperactivity items were included in the analysis.

### 2.4 Data harmonization methodology

We harmonized data by using linear equating, kernel equating, IRT and various ML based methods and compared the quality of harmonization results. But first it was important to decide which scale to use as the target scale. In the case of harmonizing the SDQ and the CBCL scale scores, there are three options: either (1) we leave the SDQ scores as they are and transform the CBCL scores in such a way they can be interpreted as SDQ items, (2) we leave the CBCL scores as they are and transform the SDQ scores to CBCL scores, or (3) we define a new scale, and we translate both SDQ and CBCL scores to that new scale. Van den Berg et al. (2014) used the option to create a new scale, but for practitioners it seems more logical to choose either the SDQ scale or the CBCL scale as the target, as these scales are already familiar to them. But which scale should be chosen as the target scale? For harmonization in daily practice, it is important to keep as much of the original information as possible. When more cases with only SDQ scores are present than children with only CBCL scores, it makes sense to leave the SDQ data as they are and find a way to transform the CBCL scores into SDQ scores. However, any large differences in the reliability of the scores should also be considered (Mansolf et al., 2022). When the CBCL scale scores are substantially more reliable than the SDQ scores, there should be a preference to leave the CBCL scores intact and find a way to translate SDQ scores into CBCL scores. With the present RAINE data set, all children had both SDQ and CBCL scores, so relative number of cases was not a consideration. We found however that the CBCL scores we slightly more reliable than the SDQ items (based on Guttman’s Lambda-2, see results), so we devised models to transform SDQ scores into CBCL scores.

For all methods we applied the same logic: we constructed a function or model that determines how to translate one scale score (SDQ) to an equivalent score on the other scale (CBCL). In all methods, the model was constructed based on a training set: one subset of the data based on a random selection of children. To check the effectiveness of each model, the model was applied to the remaining children using only the SDQ data as if the CBCL data were missing, predicting the CBCL score, and comparing it with the actual observed score.

#### 2.4.1 Equating based on distributions only

The most common traditional (non-IRT) methods are mean, linear or equipercentile equating. Those methods are focused on the test level scores and they are described in detail by . In mean equating, the scores on test B are transformed such that the transformed scores have the same mean as the scores on test A. Linear equating takes into account not only the means but also the standard deviations. A linear function is estimated that translates the scores on test A such that they have a comparable mean and standard deviation as the scores on test B. We used the ‘equate’ package from R to conduct linear equating.

When not only the means and variances of two scales are different (the first two moments), but the whole shape of the distribution looks different, it is necessary to also make the higher moments equal (i.e., skewness, kurtosis). For that we can use equipercentile equating where, after a nonlinear transformation, the scores on tests A and B have equal percentile ranks.

#### 2.4.2 Equating exploiting the single group design

Kernel equating is a more elaborate method to make the distribution of one score more like the one for another score. It also includes the possibility to use more information that is available when the scores are coming from the same individuals. In kernel equating, the scores are first converted from discrete to continuous using for example a Gaussian kernel distribution (von Davier et al., 2006; Liu and Low, 2008; Arikan and Gelbal, 2018). Kernel equating can be used in such a way that one exploits the single group design: the information of what CBCL scores go together with which SDQ scores in the same children. We used the ‘kequate’ package with the single group option (Andersson et al., 2013).

#### 2.4.3 Item response theory (IRT) and other model based approaches

The IRT approach uses the responses to the individual items, rather than the total scores. A model is used that links a participant’s response to an item to both the participant’s trait level and the item parameters of that particular item (Embretson and Reise, 2000). One commonly used model is the Generalized Partial Credit Model (GPCM; e.g., van den Berg et al., 2014; Jović et al., 2022). This model contains one discrimination and several threshold parameters for each item (Embretson and Reise, 2000). The discrimination parameter represents the capability of an item to differentiate among respondents with similar trait levels (Embretson and Reise, 2000). It is conceptually similar to a factor loading in factor analysis (van den Berg et al., 2007). The threshold parameters are defined as the point on the latent trait continuum where the response probability for two adjacent response categories is equal (Wetzel and Carstensen, 2014). Accordingly, for a 3-point scale, we have two threshold parameters, between categories 1 and 2 and between categories 2 and 3 (Uto and Ueno, 2018). For the IRT approach we used the mirt package (Chalmers, 2012) to estimate the discrimination and threshold item parameters of the GPCM. The IRT harmonization approaches focuses mainly on the items: based on the SDQ item scores, an estimate is made of an individual’s latent trait level, after which this latent trait estimate is used to make a prediction of the total score on the CBCL, conditional on the CBCL item parameters.

Apart from IRT, several other models were tried that are not traditionally using in test equating: linear and ordinal regression, support vector machines (SVM; Hearst et al., 1998; Noble, 2006; Awad and Khanna, 2015 and random forest).

Regression is a statistical technique that relates a dependent variable to one or more independent (explanatory) variables and it plays a fundamental role in statistical modelling. It is widely used in a form of linear regression where dependent variable is continuous. There is also variant of regression for predicting responses on a categorical scale, ordinal regression. Ordinal regression objective is to classify patterns using a categorical scale which shows a natural order between the labels, and in the case when the scale is ordinal, the ordering consideration improves the performance in comparison to their nominal equivalents (Gutiérrez et al., 2015). You can find more details about ordinal regression and underlying formulas in (Tutz, 2022).

A support vector machine (SVM) is a computer algorithm that learns by example to assign labels to objects. In general, a SVM is an algorithm for maximizing a particular mathematical function with respect to a given collection of data (Noble, 2006).

For more details and underlying mathematical formulas behind SVM, check Noble (2006), Hearst et al. (1998), and Awad and Khanna (2015).

Random forest (RF) is a supervised learning algorithm that combines the output of multiple randomized decision/regression trees to reach a single result by averaging them (Biau and Scornet, 2016). Random Forests can be used for either a categorical response variable as “classification” or a continuous response, referred to as “regression” (Cutler et al., 2012; Qi, 2012).

**Figure 1:**
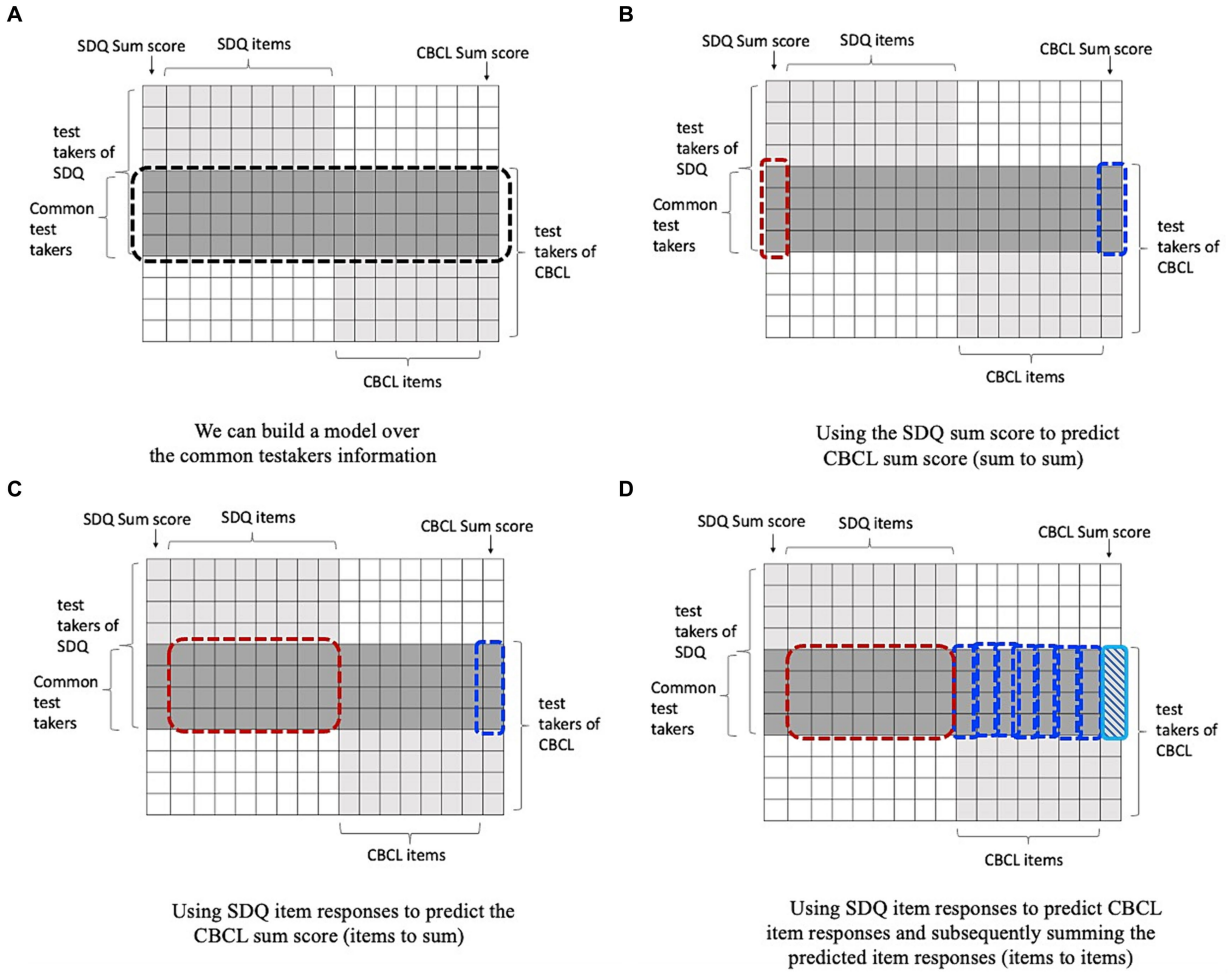
Illustration of using common persons design for harmonization. **(A)** shows the common person harmonization problem. We have two tests SDQ and CBCL; for the common person, we have the responses for both sets of items. Thus, we can train and validate a model for predicting the outcomes of one test from the other one over the information of common persons using three different ways. **(B)** shows the sum to sum method for harmonization using machine learning. **(C)** demonstrates the items to sum approach, and **(D)** depicts item to items approach.

Within these methods, there are several options on how to use them. The IRT approach tis fully focused on item level data, where the information on the SDQ items is used to make a prediction of the SDQ sum score through the latent trait level and the item parameters. In contrast, for the other methods we can choose whether to work with the raw item data or with the total scores, for both the target scale (CBCL) as the original scale (SDQ). We harmonized data in three different ways (these methods are illustrated in Figure 1):

- Using the SDQ sum score to predict CBCL sum score (sum to sum).
- Using SDQ item responses to predict the CBCL sum score (items to sum).
- Using SDQ item responses to predict CBCL item responses and subsequently summing the predicted item responses (items to items).

Note that sum to sum prediction was only realistic in the case of linear and ordinal regression, but was not sensible in the case of Random Forest and Support Vector Machine since you then have only one predictor variable.

Apart from the choice whether to work with items or total scores, there is also the choice regarding the measurement level of the target variable. Regression approaches in ML regard the target as having interval measurement level, whereas classification approaches regard the target variable as a categorical variable (nominal measurement level). In between is the option to regard the target as ordinal. We therefore applied a linear regression (using the ‘lm’ function from the R stats package) and compared it to an ordinal regression, using the ‘clm’ function for ordinal regression model (R package ‘ordinal’; Christensen, 2018). For the SVM we used both the regression and the classification version with the package ‘e1071’ (Dimitriadou et al., 2009). We used the ‘svm’ function both for classification (type = ‘C-classification’, kernel = ‘linear’) and regression (type = ‘eps-regression’, kernel = ‘linear’). For random forest ordinal classification, we used the ‘ordfor’ function (‘ordinalForest’; Hornung, 2020) and for random forest regression the ‘randomForest’ function (‘randomForest’; Liaw and Wiener, 2002).

### 2.5 Evaluating the quality of harmonization

We evaluated the quality of harmonization by comparing the scores as predicted by the models (i.e., the harmonized scores) to the observed (true) ones by computing the root mean squared error (RMSE). First, we calculated the difference between the observed and predicted scores by certain method for every participant, squared them and summed them for all participants (or data points). After that, we divided the sum with the number of data points in order to get the mean value and calculated the square root of the mean value to get RMSE.

A small RMSE represents small differences between observed and predicted scores, and therefore high-quality harmonization. To avoid overfitting and to get a realistic idea of how well the methods would work in practice, we used 5-fold cross-validation. We randomly divided our sample (1,551 participant with complete responses on all 11 CBCL and all 5 SDQ items; no missing data) into 5 subsets (folds). We used 4 folds as a training set to estimate the model, and one-fold as a test set (80% training, 20% test), predicting the CBCL data on the basis of the SDQ data. Every fold was used once as the test set. We used the RMSEs across the five folds to construct boxplots. Next to these RMSE boxplots, we used scatterplots of observed and predicted scores to further illustrate differences between the methods.

## 3 Results

The CBCL scale had a slightly higher reliability in comparison with SDQ scale (0.82 vs. 0.80). Consequently, we decided to use the SDQ scale score as the predictor and CBCL scale as the criterion.

**Figure 2:**
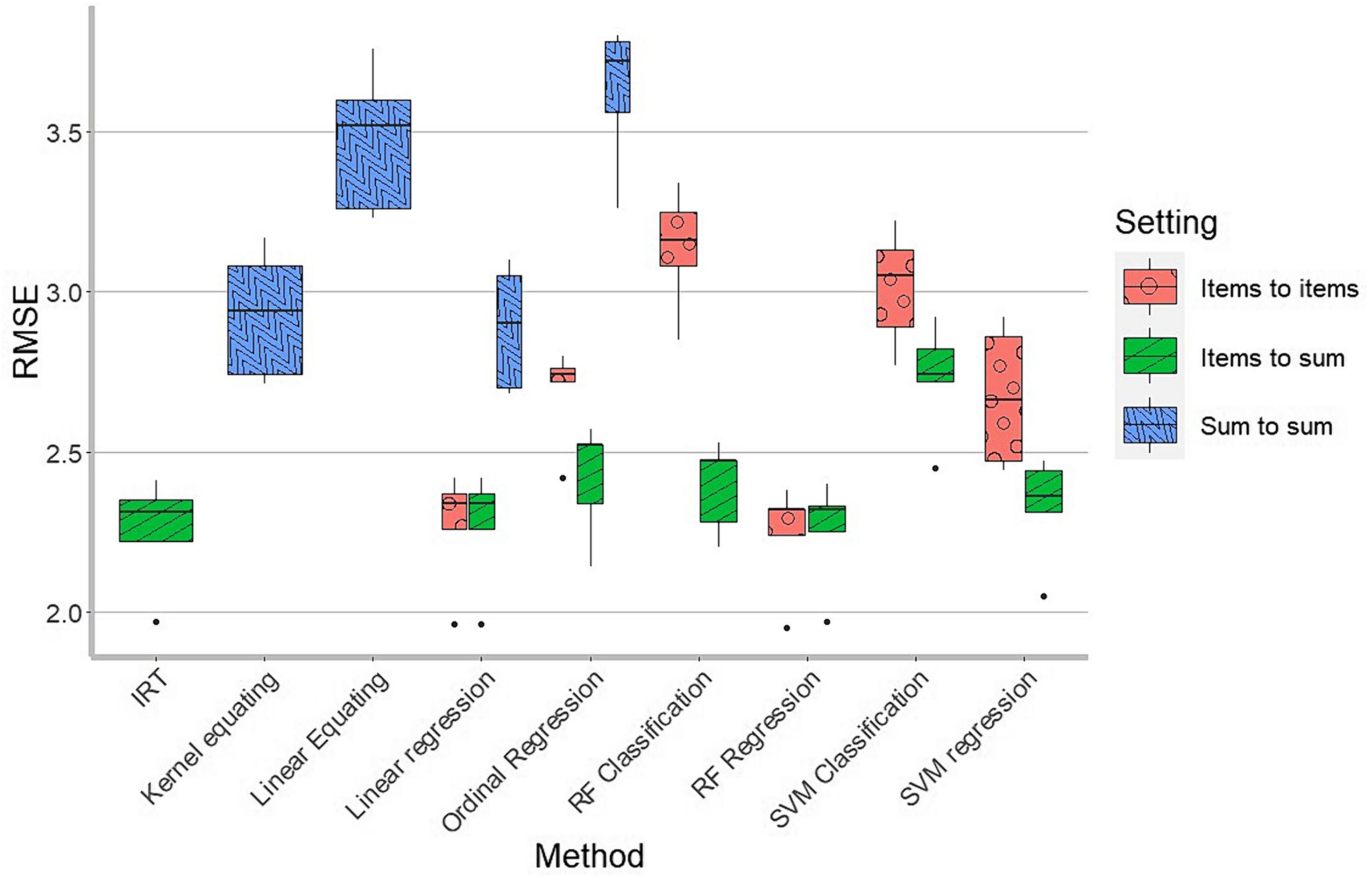
Boxplot of RMSEs in 5-fold cross-validation, as a function of harmonization method. Per method we see the spread of the RMSEs in the 5-fold cross-validation. There are in total 17 boxplots. 6 for items to items setting (Linear regression, Ordinal regression, RF classification, RF regression, SVM classification and SVM regression), 7 for items to sum setting (IRT, Linear regression, Ordinal regression, RF classification, RF regression, SVM classification and SVM regression) and 4 for sum to sum setting (Linear equating, Kernel equating, Linear regression and Ordinal regression).

The RMSEs associated with the various methods are presented in Figure 2 and Table 1. The various methods showed a large variation in performance. The overall worst performance was seen in the ordinal regression with the sum score as the predictor. The other methods that used only the SDQ sum score as predictor were also relatively poor, compared to the methods that used individual SDQ items as predictors. Overall, the items to sum options (in green) performed better than the items to items options (in red), except for linear regression and random forest regression where they showed comparable success. Overall, it seems best to use individual SDQ items to predict the CBCL scale score directly. Another pattern is that the regression approaches perform better than the classification/ordinal approaches, that is, regarding the output as interval measurement level rather than ordinal or nominal.

**Table 1:** Summary of the results for different methods and settings.

Method	Setting	Median RMSE (lower the better)	Mean RMSE	SD RMSE
IRT	Items to sum	2.31	2.25	0.17
Linear equating	Sum to sum	3.52	3.47	0.23
Kernel equating	Sum to sum	2.94	2.93	0.20
Linear regression	Items to items	2.34	2.27	0.18	Items to sum	2.34	2.27	0.18	Sum to sum	2.9	2.89	0.19
Ordinal regression	Items to items	2.74	2.69	0.15	Items to sum	2.52	2.42	0.18	Sum to sum	3.72	3.62	0.22
Random forest regression	Items to items	2.32	2.24	0.17	Items to sum	2.32	2.25	0.17
Random forest classification	Items to items	3.16	3.14	0.19	Items to sum	2.47	2.39	0.14
SVM regression	Items to items	2.66	2.67	0.22	Items to sum	2.36	2.33	0.17
SVM classification	Items to items	3.05	3.01	0.18	Items to sum	2.74	2.73	0.18

Methods with the best results (the lowest RMSE) are highlighted.

Generally, we see that the IRT method, the linear regression and the random forest regression showed the best results, with very similar RMSEs.

Kernel equating performed better than linear equating, which is to be expected since it exploits the single group design whereas linear equating only uses the means and standard deviations of the SDQ and CBCL score distributions.

**Figure 3:**
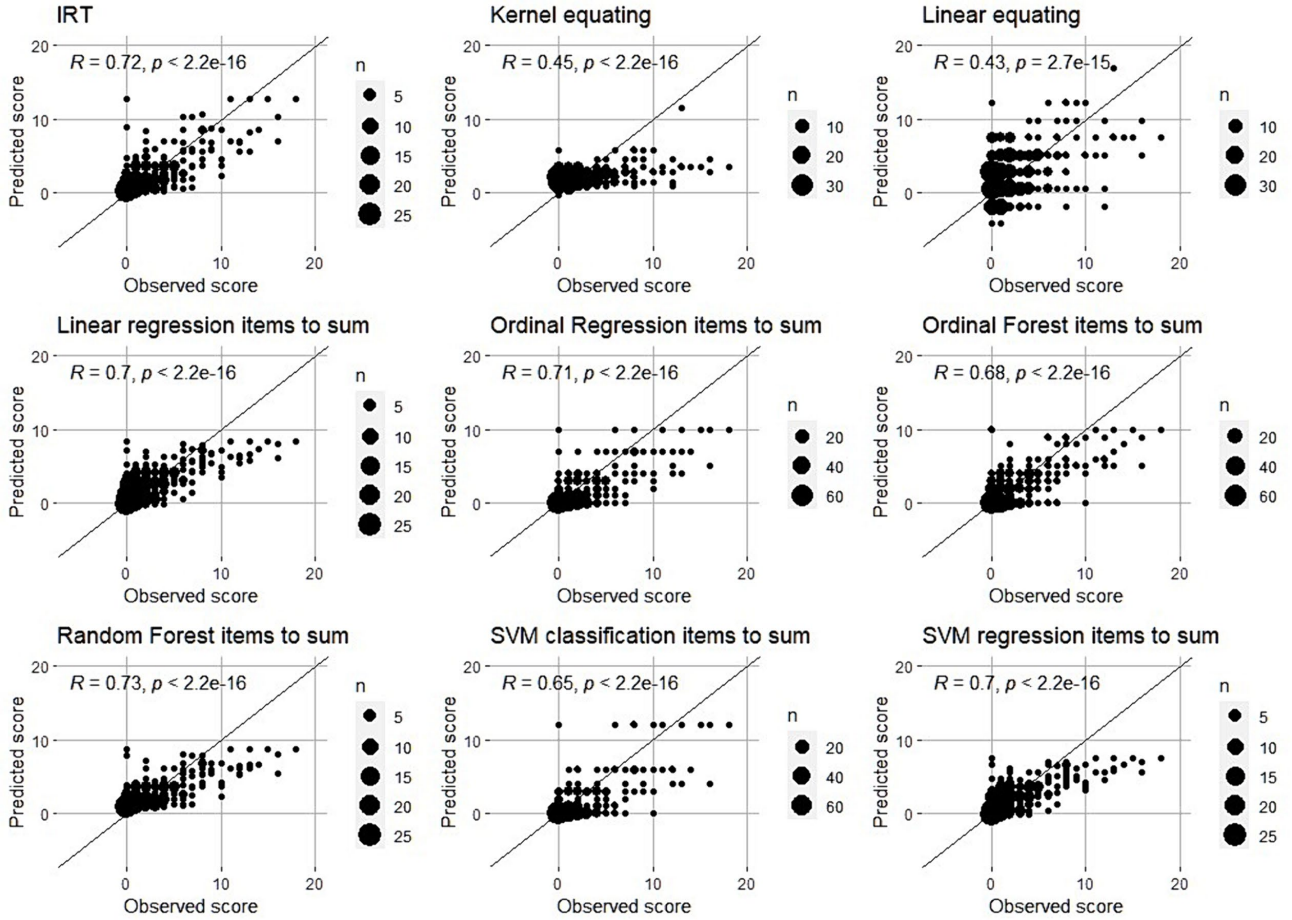
Observed vs. Predicted scores – Scatter plots. Black line is the line with intercept 0 and slope 1. In the case of perfect quality of harmonization, all points should be on that line which would mean that predicted and observed scores are exactly the same.

Figure 3 shows the relationship between predicted and observed scores for the linear and kernel equating, IRT and ML regression methods. For clarity, the line with intercept 0 and slope 1 is drawn where the dots should be in case of perfect prediction. In order to avoid presenting the mixture of 5 subsets (folds) in the same graph, we used the results from 1 fold as an example.

First thing that we can clearly see is that harmonization of SDQ scores close to 0 is more precise than harmonization of high scores (above 10). For scores close to 0, the predictions are relatively good, with only some overestimation. Scores above 10 are generally far from the perfect line and generally underestimated. It seems particularly hard to harmonize the relatively high scores. It is worth mentioning that in the case where majority of responses are skewed towards the lower side of the scale lead to underestimation of high scores which also affects RMSE values. That is, methods that underestimate the high scores more are expected to have higher RMSE.

## 4 Discussion

The aim of this paper was to harmonize ADHD scores measured by CBCL and SDQ scales. We used different data harmonization methods with different levels of complexity, different underlying theories and limitations. We compared the quality of harmonization obtained by linear and kernel equating, IRT and three different machine learning methods (regression, random Forest and SVM) by using both regression and classification approaches. The methods showed a large variety in performance. The best performing models were based on SDQ items rather than SDQ sum scores, and treated the outcome as interval measurement level (referred to as a regression approach in machine learning), rather than ordinal or nominal (classification). The IRT method, the random forest regression and the linear regression based on items showed the best overall performance in terms of RMSE.

Looking more closely at these three methods, the random forest regression and the linear regression showed very comparable patterns in the scatterplot of observed and predicted scores. The pattern was slightly different in the IRT approach with more overestimation for the low scores and less underestimation but more variability for the higher scores. It seems that the bias for the IRT model is less, but that the variance of the predictions is larger.

For all methods there was a bias in that low scores were overestimated and high scores were underestimated. This is due to three causes: impossibility of scores less than 0, regression towards the mean and lack of information on the high score end of the scale (sparsity). For instance, in the IRT approach and in the classification approaches, it is simply impossible to predict a sum score less than 0, so all misclassifications of CBCL scores of 0 are due to overestimation. In the linear regression approach, there is a natural regression to the mean since the correlation between a weighted sum of SDQ item scores and CBCL sum scores is never 100%. Because the relationship is forced to be linear, there will be overestimation on the low end of the scale and underestimation on the high end. An increase in sample size will never fix this problem. On the other hand, in the random forest approach, a nonlinear relation between the items and the outcome is possible. With increasing data on the higher end of the scale it is theoretically quite possible to get better results. In this study the number of children with high scores on attention problems/hyperactivity were relatively scarce since the sample was from the general population. Future research should look deeper into the relationship between sample size, sparsity, bias and variance for the IRT, linear regression, and random forest methods.

Machine learning methods performed better than linear and kernel equating in all the cases except for ordinal regression in sum to sum setting. The lower quality of harmonization in the case of ordinal regression in sum to sum setting could possibly be explained by the fact that we use limited amount of information (only one predictor, SDQ sum score) as in the linear and kernel equating, but on the top of it predicted scores are rounded to be on an ordinal scale in the case of ordinal regression. In that way, by rounding the scores we lose some information which is not the case with linear and kernel equating which scores are not necessarily round numbers. In the case when we use same small amount of information in different methods (linear and kernel equating and ordinal regression in sum to sum setting), losing information due to rounding the scores could make the difference in favour of method which does not round the scores and keeps more information (linear and kernel equating). That is something that would be good to pay attention to and investigate further in the future research. In all other cases, machine learning methods performed better than linear and kernel equating and were very close to IRT. That is in accordance to the findings of the previous studies. For example, Tsutsumi et al. (2021) showed that machine learning can be used for successful data harmonization (in their study they combined it with IRT), while in the study of Jiang et al. (2023) machine learning methods outperformed mean, linear and equipercentile equating. Machine learning methods are more advanced, more complex and take into account more information than the linear and kernel equating methods that are often used in the educational assessment field (i.e., item level data). Our results confirmed that machine learning has strong potential.

Because the best performing methods used item level data, it is not straightforward to construct crosswalk tables that provides researchers the information what SDQ score is equivalent with what CBCL score. Although quick and easy, we do recommend to instead use SDQ item level information to predict the equivalent CBCL, as that yields more reliable results.

We used a harmonization approach where we attempted to find a function that transforms an SDQ score into a CBCL-like score, in such a way that all scores can now be interpreted as CBCL scores. In practice that would mean that if you have several groups of children that were assessed using the CBCL and several groups of children that were assessed using the SDQ, you can keep the original CBCL scores, and only have to transform the data from the children with SDQ data. In this context, it is important to mention that we had the same sample sizes for CBCL and SDQ data, so we decided which scale to use as a predictor based on scale reliability (by using more reliable scale as a criterion to keep as much information as possible at the end). But in the case if samples are not equal and if we have larger sample size for, for example, SDQ than for CBCL, then it would make more sense to use the scale with larger sample size as a criterion in order to keep more of the original information, especially in the case if scale reliability of two scales is very similar.

Our results showed that an approach based on the raw SDQ item scores works better than using the sum score only. Moreover, based on previous research we knew that attention problems/hyperactivity scores from CBCL and SDQ scales can be harmonized successfully by using IRT (Jović et al., 2022) in the sense that the IRT unidimensional model fitted reasonably well. In this research paper we showed that for attention problems/hyperactivity the IRT approach gave the best results: it is better at predicting what the CBCL score would look like than many of the other methods. This is surprising given the fact that the CBCL attention problems subscale and the SDQ hyperactivity subscale do not fully match content-wise. You would expect that the constructs assessed with these two scales overlap, but not 100%. Both measure ADHD in a broader sense, but with different emphasis to hyperactivity and attentional problems. SDQ is focused on hyperactivity, while CBCL is more focused on attention problems. They are correlated (r = 0.43), but they do not completely overlap conceptually. In that case, one would expect the IRT model that we applied here would not be ideal as the IRT model assumed there is only one dimension underlying all SDQ and CBCL items. But here we saw that the approach works is quite robust to model violations in that the other methods performed similarly or worse.

Stevens et al. (2021) successfully harmonized total SDQ and CBCL scores by using equipercentile equating. Mansolf et al. (2022) also harmonized scales on a more general level (Internalizing, Externalizing, Total problems). They also used a single group design and the results showed that both IRT and kernel equating led to similar quality of harmonization, which was quantified as a high correlation between predicted and observed scores. It should be noted Stevens et al. (2021) and Mansolf et al. (2022) did not use a cross-validation approach (at least not within age groups) so that it is unsure to what extent there was model overfit.

We zoomed in on the more specific subscales hyperactivity and attention problems where the conceptual overlap is less precise, the scale reliabilities are lower, and consequently, the correlation between the observed scores is lower. This inevitably results in lower quality of harmonization. The correlation between observed and predicted scores was between 0,43 and 0,73, depending on the method. The unidimensionality assumption is particularly pertinent in the IRT approach we took. It would therefore not be strange to find that one of the machine learning methods would perform better as these do not have this unidimensionality assumption. In our study, machine learning methods (especially with a regression approach) led to much higher quality of harmonization than linear and kernel equating but were comparable to IRT. Potential explanation is in the similarity of the underlying mechanisms of these methods. Namely, in the regression approach, every predictor (item) is weighted and contributes to the prediction of criterion in different degree, while in the IRT approach we have discrimination parameters that are doing the same by referring to the strength of the relationship between trait level (criterion) and participants` responses on the item (predictor). There is a strong case to believe that the IRT approach can often outperform linear regression. The IRT approach takes advantage of both linear and non-linear transformations. In the first part of IRT, there is nonlinear, more or less S-shaped, translation of item scores to latent trait level while in the next step the estimated latent trait values are translated to sum score using again a nonlinear S-shaped form (depends on the exact IRT model that was used). In contrast, with linear regression there is only a strictly linear relationship between the weighted SDQ sum score and the unweighted CBCL score. We expect IRT to outperform linear regression in cases where the threshold item parameters are very different across scales. Overall, we expect that with less sparsity in the top end of the scale, the random forest approach can outperform the IRT and the linear regression approaches.

At the end, it is worth mentioning that even though the quality of harmonization conducted by linear and kernel equating methods was not very good, there are situations in which those methods are only possible methods for data harmonization. That is the case if we do not have the full data obtained by the Single Group design (Common persons) but we only have summary statistics. In addition, in the context of quality of harmonization and machine learning it is important to mention that even though our results showed that regression approach gives better results in comparison to classification, treating categorical variable as continuous and using regression approach may not always be possible.

Concluding, when harmonizing data, different methods should be tested for a particular application, making use of cross-validation to avoid overfitting. Whenever data is available from the same individuals, one should make use of either IRT or regression based machine learning methods (in the case if regression based approach is suitable) that use the items as predictors.

## Data availability statement

The data analyzed in this study is subject to the following licenses/restrictions: Data is not publicly available because it is owned by Raine Study and permission for getting and analyzing it should be obtained from them. Requests to access these datasets should be directed to andrew.whitehouse@telethonkids.org.au.

## Ethics statement

The studies involving humans were approved by the broader Raine Study and have ethics approval from The University of Western Australia Human Research Ethics Committee. The studies were conducted in accordance with the local legislation and institutional requirements. Written informed consent for participation was not required from the participants or the participants' legal guardians/next of kin in accordance with the national legislation and institutional requirements.

## Author contributions

MJ: Conceptualization, Methodology, Visualization, Writing – original draft, Writing – review & editing. MH: Conceptualization, Methodology, Supervision, Writing – review & editing. AW: Resources, Writing – review & editing. SB: Conceptualization, Methodology, Supervision, Writing – review & editing.
